# Nitazoxanide Exhibits Schistosomicidal Activity Against Schistosomula and Adult *Schistosoma mansoni* and Influences Praziquantel-Associated Outcomes

**DOI:** 10.1007/s11686-026-01269-2

**Published:** 2026-05-13

**Authors:** Cícero Jádson Da Costa, Juliana Ellen de Melo Gama, Alex José de Melo Silva, Karina Lidianne Alcântara Saraiva, Christina Alves Peixoto, Roni Evêncio de Araújo, Carlos André Laranjeira Miranda Filho, Gustavo Henrique Aires Albuquerque, Danielle Maria Nascimento Moura, Sheilla Andrade De Oliveira

**Affiliations:** 1 Department of Immunology, Laboratory of Immunopathology and Molecular Biology, Oswaldo Cruz Foundation (Fiocruz), Aggeu Magalhães Institute, Pernambuco, Brazil; 2 Oswaldo Cruz Foundation (Fiocruz), Aggeu Magalhães Institute, Technological Platforms Network, Electron Microscopy Platform, Pernambuco, Brazil; 3 Department of Microbiology, Oswaldo Cruz Foundation (Fiocruz), Aggeu Magalhães Institute, Ultrastructure Laboratory of the Aggeu Magalhães Institute, Pernambuco, Brazil

**Keywords:** *Schistosoma mansoni*, Anthelmintic drugs, Murine experimental model, Ultrastructure, Drug repositioning

## Abstract

Schistosomiasis remains as a parasitic disease with major morbidity, ongoing transmission, and economic burden, particularly in tropical regions. Praziquantel is the first-line treatment but has limited efficacy against juvenile stages of the parasite. In this context, this study assessed the repurposing potential of anthelmintics against *Schistosoma mansoni*, focusing on the schistosomicidal activity of nitazoxanide (NTZ), levamisole (LVZ), and thiabendazole (TBZ). In vitro, NTZ exhibited a significant schistosomicidal activity, emerging as a repurposing candidate. In contrast, LVZ and TBZ showed no significant schistosomicidal activity. NTZ was active from 40 µg/mL, affecting schistosomula and adult worms in a dose-dependent manner. Ultrastructural analysis revealed wrinkling of the oral and ventral suckers and tegumental alterations in male worms, including shortening of ridges near the tubercles and the presence of multiple small protrusions. In vivo, NTZ showed partial efficacy, with 57.14% reduction in adult worm recovery, whereas PZQ achieved complete elimination. Infected animals presented a mean of 541.86 ± 273.13 viable eggs/cm^2^, while treatment with nitazoxanide (205 mg/kg) or praziquantel (400 mg/kg) reduced these values to 247.98 ± 109.68 and 144.92 ± 133.46 eggs/cm^2^, respectively. Combined administration of NTZ and PZQ resulted in the greatest reduction in viable egg counts, with a mean of 96.75 ± 49.55 eggs/cm^2^. Although praziquantel resistance has been reported, in this study it fully eliminated adult worms, while nitazoxanide showed lower but relevant activity against both schistosomula and adult stages. These findings confirm the efficacy of praziquantel against adult worms and highlight therapeutic potential of nitazoxanide, underscoring the need for further bioavailability studies.

**Graphical Abstract:**

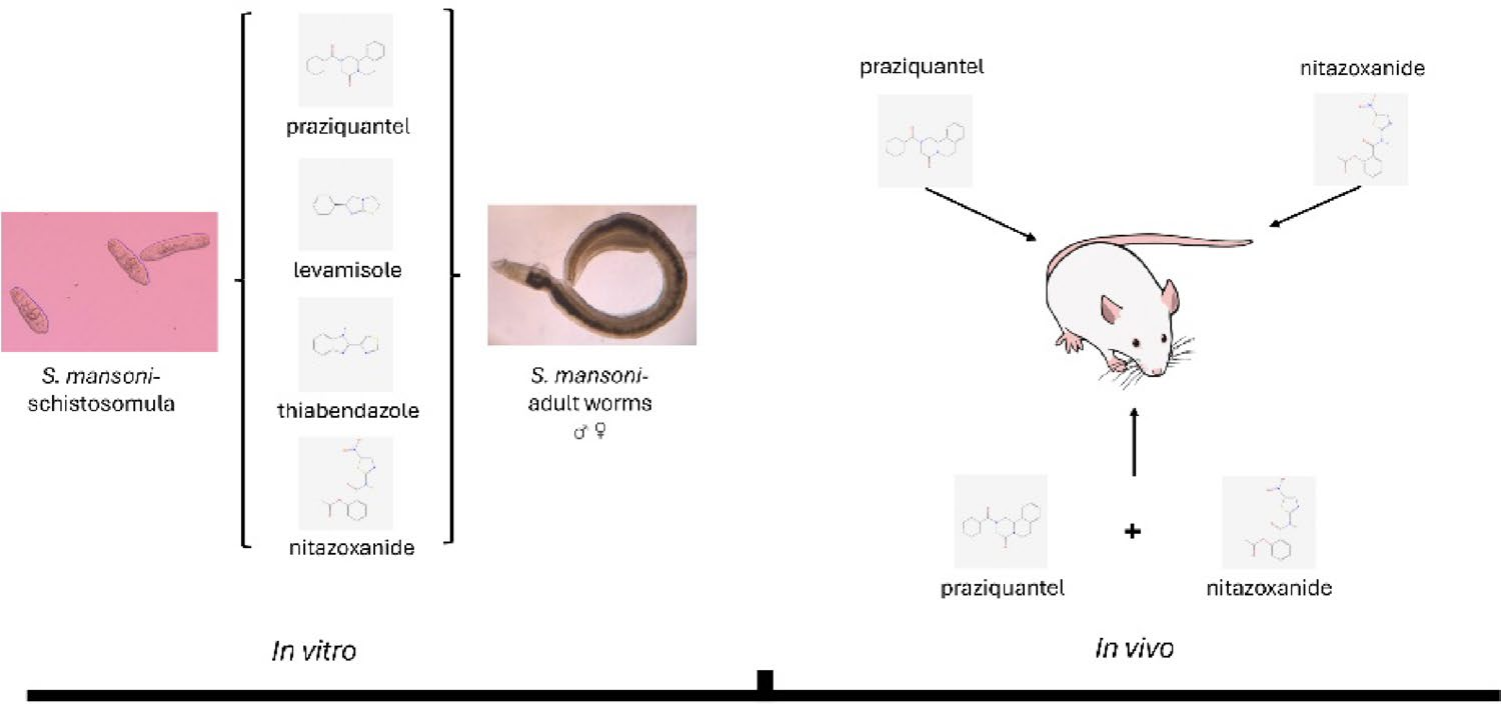

## Introduction

Praziquantel (PZQ), a pyrazinoisoquinoline derivative, is widely recognized as the first-line drug for the treatment of schistosomiasis due to its efficacy against different *Schistosoma* species in their adult forms. However, the effectiveness of praziquantel is limited against schistosomula and juvenile worms, and cases of temporary resistance have been reported in in vitro studies conducted in Africa [1–6]. A major factor contributing to this therapeutic gap is that praziquantel, despite inducing contractile paralysis and tegumental disruption across developmental stages, triggers loss of mitotic activity only in adult worms. Moreover, the parasite’s developmental stages display distinct tissue tropism: adults predominantly reside in mesenteric and hepatic venous plexuses; whereas juveniles transit through the skin, systemic circulation, and pulmonary parenchyma before reaching the liver to complete maturation and migrate to the portomesenteric vasculature [7–10] In endemic regions, individuals infected with *Schistosoma mansoni* often harbor both juvenile and adult worms concurrently due to sustained exposure to infective stages [1]. Thus, due to the therapeutic gap left by PZQ in the treatment of *Schistosoma* spp. larvae, recent studies have focused on investigating natural compounds and drugs with potential anthelmintic activity [2].

Drug repositioning has emerged as an effective strategy to accelerate the development of new therapies, reducing costs and time while leveranging established knowledge of safety and pharmacokinetics. Recent examples are: the anticancer agent Salirasib, which demonstrated antimalarial activity (11]; clofazimine, originally developed for leprosy, which showed efficacy against *Trypanosoma cruzi*,* Babesia*, and *Cryptosporidium*, the latter currently under evaluation in phase IIa clinical trials in HIV-coinfected patients [12–15]; and the widely used antimalarial mefloquine, which displayed antitumor effects in colorectal cancer models [16]. Furthermore, drug combinations such as benznidazole and chloroquine have been shown to enhance trypanocidal activity in vivo, allowing dose reduction and minimizing toxicity [17–18]. Although most of these advances have been reported in protozoan and other parasitic diseases, they underscore drug repositioning as an innovative and highly promising approach for schistosomiasis [19]. In endemic areas, combining drugs with complementary mechanisms of action may offer a broader therapeutic coverage and mitigate risks associated with resistance and the limited efficacy of PZQ against the parasite’s juvenile forms [3, 5].

Among the candidates for schistosomiasis treatment, three anthelmintic drugs stand out: Nitazoxanide (NTZ), a thiazolide derivative whose active metabolite tizoxanide drives most of its biological activity, has emerged as a broad-spectrum candidate for schistosomiasis control. Beyond its initial indication against *Giardia* and *Cryptosporidium*, NTZ exhibits antiviral, antibacterial, antiprotozoal, and anthelmintic activities [20–25]. Its mechanisms include the inhibition of pathogen replication and protein processing, as well as interference with key metabolic and detoxification pathways. [26–27]. NTZ has also been reported to exert a pleiotropic antimicrobial effect by collapsing membrane potential and disturbing intracellular pH homeostasis, which supports its therapeutic versatility [28–32]. In helminths, NTZ has been shown to trigger profound metabolic shifts associated with energy stress [33] and to induce ultrastructural tegumental damage [34]. In addition to its direct antipathogen effects, NTZ has also been reported to display immunomodulatory properties in human immune cells, including effects on cytokine production and immune cell polarization [35].

Thiabendazole (TBZ), the first benzimidazole derivative described as an anthelmintic agent, was initially proposed to act by inhibiting key metabolic enzymes, such as fumarate reductase and malate dehydrogenase [36–37]. Subsequent studies demonstrated that TBZ binds selectively to β-tubulin, blocking microtubule polymerization, disrupting cellular architecture, and ultimately leading to parasite death [38]. In addition to its established anthelmintic and antifungal properties, TBZ has gained attention in cancer research due to its capacity to inhibit angiogenesis, functioning as a vascular-disrupting agent [39–44].

Levamisole (LVZ), a thiazole derivative, is widely used against *Trichuris trichiura*, *Ascaris lumbricoides*, and hookworm infections (45–46]. As a cholinergic anthelmintic, it acts through the selective activation of acetylcholine-gated ion channels [46]. Its immunomodulatory properties have stimulated investigations in cancer research (47–48]. When used as an adjuvant or in combination with 5-fluorouracil (5-FU) for stage III colon cancer, LVZ enhances the antitumor efficacy of chemotherapy by reducing cancer cell proliferation and inducing apoptosis. These effects that have been associated with the activation of apoptosis-related signaling pathways, including ERK phosphorylation and increased expression of death receptor 4 (DR4) [49–50]. These effects have been observed in lung cancer models in vitro and in vivo [48, 50]. Phase III clinical trials are currently evaluating LVZ in combination with arginine hydrochloride for the treatment of advanced hepatocellular carcinoma (HCC) (NCT03950518) and advanced intrahepatic cholangiocarcinoma (NCT03940378) [51]. Additionally, co-administration of LVZ with the hepatitis B vaccine in HIV-infected patients improved immune responses and antibody titers [52].

In this context, the present study evaluated the efficacy of these three anthelmintic drugs, individually and in combination with praziquantel, using in vitro and experimental infection models of *Schistosoma mansoni*. Through parasitological and ultrastructural analyses, this study generated data that may contribute to optimizing therapeutic strategies against *S. mansoni*, providing insights for the development of more effective treatment alternatives with important implications for public health in endemic regions.

## Materials and Methods

### Parasites

This study used the LE strain of *Schistosoma mansoni* (Belo Horizonte, Minas Gerais, Brazil), obtained from *Biomphalaria glabrata* snails bred in the Malacology Laboratory at the Aggeu Magalhães Institute/IAM-Fiocruz, described below.

#### Obtention of Schistosomula

To obtain cercariae, snails were placed in autoclaved water and exposed to light and heat stimulation. After one hour, the water containing cercariae was transferred to a sterile conical tube and stored at 4 °C for 30 min. The cercarial suspension was then subjected to four centrifugation cycles at 1932 × g for 3 min each. The resulting pellet was resuspended in DMEM High Glucose culture medium (12100-038; Gibco, Grand Island, USA) supplemented with 1% antibiotic/antimycotic solution (P4333; Sigma-Aldrich, Sain Louis, USA). Mechanical transformation of cercariae into schistosomula was performed as previously described Frahm et al., 2019 [53] and Tekwu et al., 2016 [54] using alternating cycles of centrifugation and vortexing until the larval tails were completely detached. Schistosomula were then placed in 96-well plates, with each well containing approximately 100–150 parasites, incubated in DMEM High Glucose medium supplemented with 1% antibiotic/antimycotic solution and 10% fetal bovine serum (FBS) at 37 °C with 5% CO₂, and maintained overnight to complete the transformation process. For each condition, experiments were conducted in two independent biological replicates, each performed in technical triplicate.

#### Adult Worms for in vitro Analysis

Adult *S. mansoni* worms used in the in vitro assays were obtained by infecting *Swiss mice (Mus musculus*) with 120 cercariae, as previously described by De Oliveira et al., 2018 [55], an experimental approach chosen to ensure sufficient parasite recovery for subsequent analyses. Briefly, mice were placed in containers with a minimal amount of warm water to stimulate defecation and urination and were then individually transferred to aerated, wide-mouthed glass flasks containing sufficient water to cover their paws and tails. Cercariae were added to the water prior to exposure, and animals remained in the flasks for 1 h under direct light to facilitate percutaneous penetration. Infection was confirmed 45 days post-infection by detecting *S. mansoni* eggs in feces using the Hoffmann technique. At 50 days post-infection, mice were anesthetized by intraperitoneal injection of ketamine hydrochloride (100–200 mg/kg) combined with xylazine hydrochloride (5–16 mg/kg) and euthanized for worm recovery [56]. All worms were recovered by perfusion, washed in high-glucose DMEM, and transferred to 96-well culture plates containing 300 µL of the same medium supplemented with 1% antibiotic/antimycotic solution and 10% FBS. For each drug concentration, experiments were conducted in technical duplicates for the three groups analyzed (male, female, and male–female pairs) and repeated in two independent biological replicates.

### Determination of Schistosomula Viability

#### Optical Microscopy

For the optical viability assay, the schistosomula were incubated in DMEM High Glucose medium supplemented with 1% antibiotic/antimycotic solution and 10% FBS and exposed to the drugs NTZ, LVZ, and TBZ at concentrations of 100 µg/mL, 80 µg/mL, 40 µg/mL, and 20 µg/mL, dissolved in DMSO. Treatment effects were evaluated at 3 h, 24 h, 48 h, and 72 h, and compared with the following to experimental controls:

- **Control 1 (CN1)**: Schistosomula in DMEM High Glucose medium, supplemented with 1% antibiotic/antimycotic solution and 10% FBS.
- **Control 2 (CN2)**: Schistosomula in DMEM High Glucose medium, supplemented with 1% antibiotic/antimycotic solution and 10% FBS and 1.6% DMSO.
- **Control 3 (CN3)**: Schistosomula in DMEM High Glucose medium, supplemented with 1% antibiotic/antimycotic solution and 10% FBS, treated with 3 µg/mL PZQ, a concentration known to exhibit schistosomicidal activity against adult worms, dissolved in 1.6% DMSO.

The viability of schistosomula was assessed by optical microscopy using a Nikon TE-2000 inverted microscope. The scoring system was adapted from Frahm et al. (2019) [53] and was based on phenotypic changes (motility, morphology, and granularity). A viability score ranging from 3 to 0 was assigned. All assessments were conducted in the two independent biological replicates described above.

- **3**: Active schistosomula without morphological alterations, regular smooth contractions, no blebs, a smooth outer surface, and no granulation, with clear visualization of internal structures under bright field microscopy.
- **2**: Reduced motility and/or increased uncoordinated activity, slight granularity, and an intact tegument with mild deformations.
- **1**: Severe reduction in motility and/or a rough outer tegument with the presence of blebs.
- **0**: Dead parasites, no movement, heavy granulation, blurred outline, rough tegument, and marked blebbing.

For each time point, the mean viability score of schistosomula exposed to each drug concentration was calculated and directly compared with the mean scores of the control groups, allowing quantitative estimation of the schistosomicidal activity. The results were interpreted based on the observed drug-induced phenotypic alterations and their corresponding viability scores.

#### Fluorescence-based Viability Assessment

Fluorescence emission pattern was used to assess the viability of schistosomula in a comparative manner. As described in the previous section, the assay included the following controls (PZQ 3 µg/mL, DMSO 1.6%, and DMEM High Glucose culture medium supplemented with 1% antibiotic/antimycotic solution and 10% FBS, as well as the following treatments (NTZ at 100, 80, and 40 µg/mL, TBZ and LVZ at 100 µg/mL). Following treatment, plates containing the NTS were centrifuged at 1000 × g for 5 min, and 16 µL of a solution containing ethidium bromide and acridine orange (100 µg/mL) was added. After 2 min, fluorescence emission was recorded under the conditions described by Smith et al. (2012) [57]. Images were captured using a LEICA DMi8 microscope equipped with FITC filters (Excitation: 460–500 nm, Emission: 541–551 nm) to detect acridine orange fluorescence and RHOD filters (Excitation: 541–551 nm, Emission: 565–605 nm) for ethidium bromide fluorescence. Differential interference contrast (DIC) microscopy was used to visualize non-fluorescent parasites.

### Susceptibility of Adult *S. mansoni* Worms to Drug Treatment in vitro

To obtain adult *S. mansoni* worms, infected Swiss mice (*Mus musculus*) were anesthetized via intraperitoneal injection of ketamine hydrochloride (100–200 mg/kg) combined with xylazine hydrochloride (5–16 mg/kg), following the conditions described by Bogdanske (2010) [56]. Worms were recovered by perfusion, washed in high-glucose DMEM, and transferred to 96-well culture plates containing 300 µL of the same medium supplemented with 1% antibiotic/antimycotic solution and 10% FBS. For each drug concentration evaluated, three wells were prepared in duplicate: one well containing four male worms, one well containing four female worms, and one well containing two male–female pairs. All assays were conducted in technical duplicates for the three groups analyzed (male, female, and male–female pairs) and repeated in two independent biological replicates. The plates were incubated at 37 °C in a humidified atmosphere with 5% CO₂, under conditions similar to those described by De Oliveira et al. (2018) [55]. Following a 24-hour adaptation period, control wells were prepared containing either medium alone or medium with PZQ 3 µg/mL or DMSO 1.6% at the respective concentrations. The drugs NTZ, TBZ, and LVZ were tested at concentrations of 100, 80, 40, 20, 10, and 5 µg/mL. Parasites were cultured for six days and evaluated every 24 h by optical microscopy. The analyses included oviposition and mortality, along with application of the motility–morphology scoring system described in Sect. 2.2.1 to characterize the parasites phenotypic status.

### Ultrastructural Evaluation of *Schistosoma mansoni* After Treatment with NTZ

For the ultrastructural analysis of NTZ-treated worms, a concentration of 10 µg/mL was used, with the drug dissolved in DMSO and incubated in DMEM High Glucose medium supplemented with 1% antibiotic/antimycotic solution and 10% FBS. Controls included worms incubated in the same medium containing 1.6% DMSO and worms treated with 3 µg/mL PZQ, which was also dissolved in DMSO and added to the same medium. All groups were incubated at the same 15-minute interval. After in vitro treatment, parasites were fixed in a solution containing 2.5% glutaraldehyde, 4% formaldehyde, and 0.1 M cacodylate buffer (pH 6.8) at room temperature and then stored at 4–8 °C for 24 h. Post-fixation was performed using 2% osmium tetroxide (OsO₄) in 0.1 M cacodylate buffer (pH 6.8) for 60 min in the dark at room temperature. Samples were subsequently washed, dehydrated through a graded ethanol series (15-minute steps), subjected to critical-point drying using liquid CO₂ (CPD030, BAL-TEC), and coated with colloidal gold. Images were acquired using a scanning electron microscope (JSM-5600LV, JEOL).

### In vivo Treatment

*BALB/c* mice (*Mus musculus*) aged 20–30 days were infected with 80 *Schistosoma mansoni* cercariae per animal, following the procedure described by De Oliveira et al. (2019) [58]. Infection conditions were similar to those described in Sect. 2.1.1, differing only in the number of cercariae and the mouse strain used.

A total of 44 female mice were used in the experiment. The uninfected group (Group I) included five animals, which were maintained throughout the entire experimental period. The remaining 39 animals were infected, and infection was confirmed 45 days post-exposure using the Hoffmann method (1934) [59]. Fifty days post-infection, treatments were administered orally, with the drugs dissolved in saline. The animals were distributed into the following experimental groups: Group II - infected and untreated (*n* = 7); Group III - treated with 400 mg/kg PZQ (single oral dose) (*n* = 4); Group IV - treated with 205 mg/kg NTZ (administered orally once daily for three consecutive days) (*n* = 7); and Group V - treated with a combination of 400 mg/kg PZQ (single dose) and 205 mg/kg NTZ (once daily for three consecutive days) (*n* = 5).The dosing regimens used in the murine model were selected based on previously reported experimental schistosomiasis studies and adjusted according to interspecies dose conversion using body surface area scaling (human equivalent dose approach) [60].

Animals were monitored throughout the experiment, and 15 days after treatment completion they were euthanized for collection of small intestine fragments for subsequent evaluation using the Oogram technique.

Differences between the initial number of animals and the final number per group were due to exclusion of non-infected animals, pre-treatment losses during infection and treatment-related mortality. Thus, the final sample size reflects anticipated loss in in vivo experiments, minimizing potential bias in the analyses.

### Parasitological Evaluation After Treatment

Parasitological evaluation was performed by comparing worm recovery from the portal venous system and the mesenteric vasculature between treated and untreated animals. The technique adopted followed the method described by Laranjeira Miranda Filho (2022) [61]. Oogram evaluation followed the classification proposed by Pellegrino and Faria (1965) [62], in which eggs were categorized as viable immature eggs (stages 1 to 4 of embryonic development) viable mature eggs, and nonviable eggs (calcified, with retracted, semi-transparent miracidium).

The efficacy of treatment with praziquantel, nitazoxanide, and their combination was determined by the percentage reduction In the parasite burden in each treated group using the following equation: Reduction of ${\mathrm{worms}}\left( \% \right) = \frac{{(C - T)}}{C}*100$, where *C* is the number of worms recovered from the control group and *T* is the number of worms recovered from the treated group.

### Statistical Analysis

Statistical analyses were performed using the R programming language 4.5.0 (2025-04-11) within the RStudio environment 2025.05.0 + 496. Normality was evaluated within each group using the Shapiro–Wilk test. Homogeneity of variances (homoscedasticity) was tested using Levene’s test. We conducted a non-parametric Kruskal–Wallis using Dunn’s post hoc test with Bonferroni adjustment. Statistical significance was set at *p* < 0.05 for all tests.

### Ethical Considerations

This study was approved and conducted in accordance with the Ethics Committee on Animal Use of the Aggeu Magalhães Institute/*Fundação Oswaldo Cruz* (IAM/FIOCRUZ), under protocol nº. 149/2019.

## Results

### Nitazoxanide Demonstrates Antiparasitic Effects on Schistosomula and Adult Worms and Significantly Inhibits Oviposition in vitro

The evaluation of the viability and morphology of *S. mansoni* newly transformed schistosomula by optical microscopy demonstrated that NTZ exhibited dose- and time-dependent activity, with effects observed even at the lowest concentrations. The response was characterized by reduced motility and/or tegumental damage in schistosomula at 20 µg/mL, and a marked reduction in motility and/or tegumental damage at 40 µg/mL. At higher concentrations (100 µg/mL), NTZ induced 100% parasite mortality within the first 3 h of incubation. In contrast, praziquantel, thiabendazole, and levamisole promoted only reduced motility and tegumental alterations, without induction of significant lethality (Table 1).

**Table 1:** Evaluation of the viability and morphology of schistosomula of *Schistosoma mansoni* after treatment with nitazoxanide (NTZ), thiabendazole (TBZ), levamisole (LVZ) and praziquantel (PZQ) by optical microscopy

Drugs												
^Nitazoxanide (NTZ)^	3 h			24 h			48 h			72 h		
100 µg/mL	0	0	0	–	–	–	–	–	–	–	–	–
80 µg/mL	0	0	0	–	–	–	–	–	–	–	–	–
40 µg/mL	1	1	1	0	0	0	–	–	–	–	–	–
20 µg/mL	2	2	2	1	1	1	1	1	1	1	1	1
Controls												
Praziquantel (PZQ- 3µg/mL)	2	2	2	2	2	2	2	2	2	2	2	2

Drugs												
Thiabendazole (TBZ)	3 h			24 h			48 h			72 h		
100 µg/mL	3	3	3	1	1	1	1	1	1	1	1	1
80 µg/mL	3	3	3	2	2	2	2	2	2	1	1	1
40 µg/mL	3	3	3	3	3	3	3	3	3	2	2	2
20 µg/mL	3	3	3	3	3	3	3	3	3	3	3	3
Controls												
DMSO (1.66%)	3	3	3	3	3	3	3	3	3	3	3	3

Drugs												
Levamisole (LVZ)	3 h			24 h			48 h			72 h		
100 µg/mL	1	1	1	1	1	1	1	1	1	1	1	1
80 µg/mL	2	2	2	1	1	1	1	1	1	1	1	1
40 µg/mL	3	3	3	2	2	2	2	2	2	1	1	1
20 µg/mL	3	3	3	3	3	3	3	3	3	2	2	2
Controls												
Culture medium	3	3	3	3	3	3	3	3	3	3	3	3

Score

0 Dead schistosomula

1 Schistosomula with marked reduction in motility and/or tegumental damage

2 Schistosomula with reduced motility and/or tegumental damage

3 Schistosomula without morphological changes (translucent) and with motility.

**-** Evaluation suspended due to mortality in the previous timepoint.

Dose- and time-dependent effects of NTZ, showing reduced motility and tegumental damage at low concentrations and early lethality at higher concentrations, compared with PZQ, TBZ, and LVZ.

**Fig. 1:**
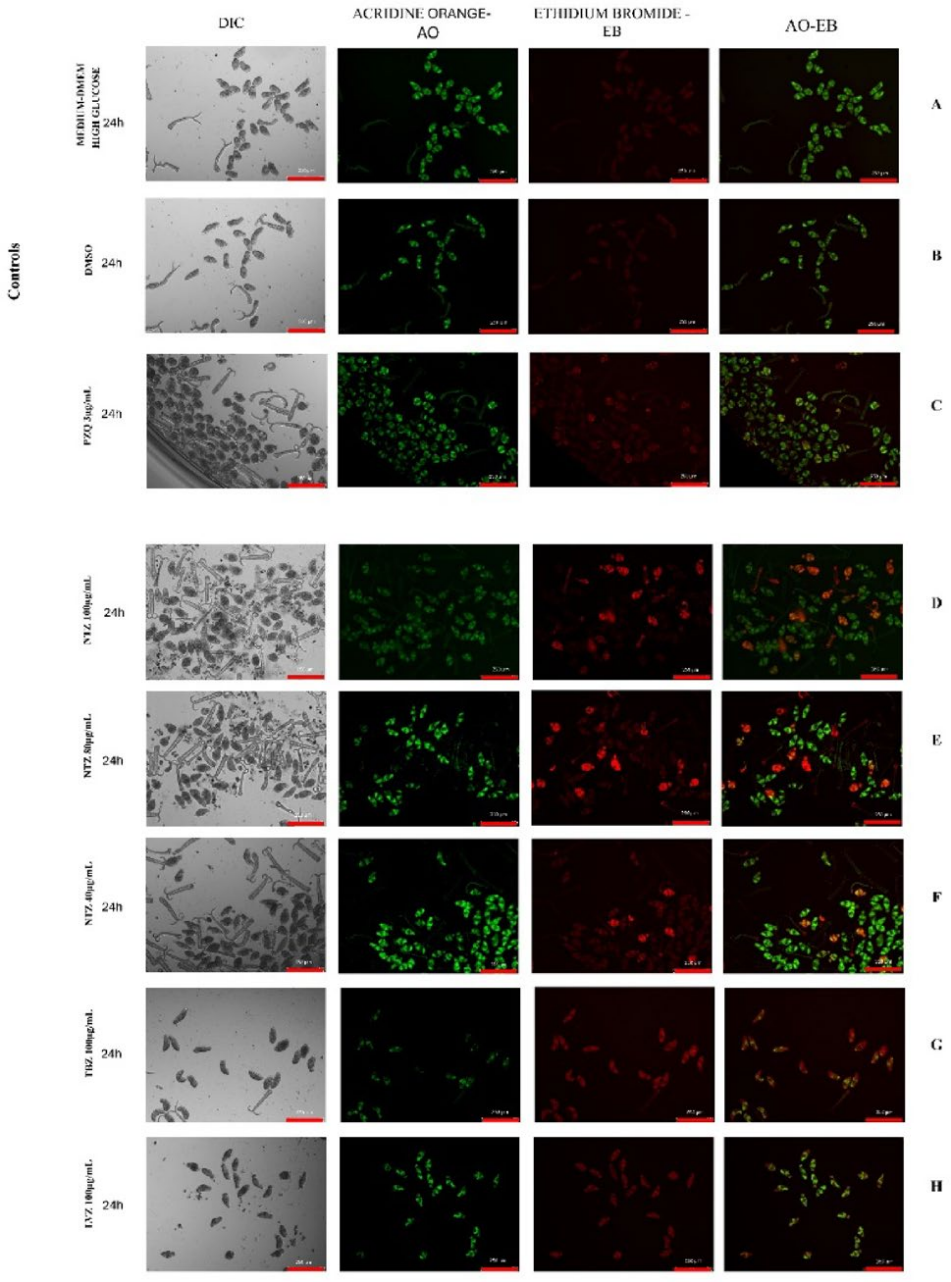
Fluorescence assay with acridine orange (AO) and ethidium bromide (EB) for viability analysis of schistosomula after treatment with nitazoxanide, thiabendazole, levamisole and praziquantel. Note: The schistosomula can be visualized in the first column by differential interference contrast (DIC). In the second column, the FITC filter shows fluorescence resulting from the permeability of acridine orange. In the third column, the fluorescence results from the permeability of ethidium bromide detected by the RHOD filter, and the fourth column shows the overlay of both captures from the two filters, FITC and RHOD. The first three lines correspond to the control groups (1st row: DMEM high glucose medium + 10% FBS; 2nd row: (1.6% DMSO; 3rd row: praziquantel 3 µg/mL). Lines 4, 5, and 6 show the schistosomula treated with nitazoxanide at 100 µg/mL, 80 µg/mL, and 40 µg/mL, respectively. Lines 7 and 8 show the schistosomula treated with thiabendazole 100 µg/mL and levamisole 100 µg/mL, respectively. All the photos were taken with the same microscope settings

Fluorescence analysis corroborated the optical microscopy findings, suggesting that the reduction in schistosomula viability upon NTZ treatment was associated with compromised membrane integrity. This was evidenced by increased ethidium bromide permeability, indicating necrotic effects (Fig. 1D, E e F). In contrast, thiabendazole (Fig. 1G), levamisole (Fig. 1H), and praziquantel (Fig. 1C) caused a less intense fluorescence signal for ethidium bromide when compared to NTZ; with no significant tegumental damage observed that would permit ethidium bromide penetration, suggesting a lack of pronounced necrotic effects.

It is important to note that during the mechanical transformation of cercariae into schistosomula, a small proportion of parasites may experience structural damage. To ensure the reliability of the results, parasite integrity was assessed 24 h post-transformation by evaluating motility prior to drug exposure, thereby minimizing potential interference from the transformation procedure. All experiments were performed in two independent biological replicates.

Consistent with the results observed in schistosomula, nitazoxanide exhibited the most pronounced schistosomicidal activity against the adult stage in vitro, except for praziquantel, which maintained high efficacy. At concentrations of 100, 80, and 40 µg/mL, NTZ induced adult worm mortality starting from the first timepoint (24 h) onwards (Table 2). Exposure of worms to NTZ at concentrations of 20 µg/mL and 10 µg/mL resulted in noticeable impairment of motility, and by the last timepoint (144 h), only female worms exhibited slight cephalic spasm-like movements. At the lowest concentration tested (5 µg/mL), NTZ reduced motility and completely inhibited oviposition. A consistent feature observed across all concentrations was an increased tegumental granulation, which was more pronounced at higher doses.

Similar results were observed in worms treated with praziquantel (Table 2). In contrast, thiabendazole and levamisole did not cause motility reduction or morphological alterations (Table 2).

**Table 2:** Evaluation of the in vitro schistosomicidal activity of nitazoxanide, thiabendazole and levamisole in adults worms

*Schistosoma mansoni*- adults worms									
Drugs	24 h			48 h			72 h		
	♂	♀	♂ + ♀	♂	♀	♂ + ♀	♂	♀	♂ + ♀
Nitazoxanide (NTZ)									
100 µg/ mL	0	0	0	–	–	–	–	–	–
80 µg/mL	0	0	0	–	–	–	–	–	–
40 µg/mL	0	0	0	–	–	–	–	–	–
20 µg/mL	1	1	1	1	1	1	1	1	1
10 µg/mL	2	2	2	1	1	1	2	2	2
5 µg/mL	2	2	2	2	2	2	2	2	2
Thiabendazole (TBZ)									
100 µg/ mL	2	2	2	2	2	2	0	2	2
80 µg/mL	2	2	2	2	2	2	2	2	2
40 µg/mL	2	2	2	2	2	2	2	2	2
20 µg/mL	2	2	2	2	2	2	2	2	2
10 µg/mL	3	3	3	3	3	3	3	3	3
5 µg/mL	3	3	3	3	3	3	3	3	3
Levamizole (LVZ)									
100 µg/ mL	3	3	3	3	3	3	3	3	3
80 µg/mL	3	3	3	3	3	3	3	3	3
40 µg/mL	3	3	3	3	3	3	3	3	3
20 µg/mL	3	3	3	3	3	3	3	3	3
10 µg/mL	3	3	3	3	3	3	3	3	3
5 µg/mL	3	3	3	3	3	3	3	3	3
Praziquantel (PZQ)									
3,0 µg/mL	0	0	0	–	–	–	–	–	–

*Schistosoma mansoni*- adults worms										
Drugs	96 h			120 h			144 h			Oviposition
	♂	♀	♂ + ♀	♂	♀	♂ + ♀	♂	♀	♂ + ♀	
Nitazoxanide (NTZ)										
100 µg/ mL	–	–	–	–	–	–	–	–	–	NO
80 µg/mL	–	–	–	–	–	–	–	–	–	NO
40 µg/mL	–	–	–	–	–	–	–	–	–	NO
20 µg/mL	0	1	1	–	1	1	–	1	1	NO
10 µg/mL	1	1	1	2	2	2	1	1	1	NO
5 µg/mL	2	2	2	2	2	2	2	2	2	NO
Thiabendazole (TBZ)										
100 µg/ mL	–	2	2	–	2	2	–	2	2	NO
80 µg/mL	2	2	2	2	2	2	2	2	2	YES
40 µg/mL	2	2	2	2	2	2	2	2	2	YES
20 µg/mL	2	2	2	2	2	2	2	2	2	YES
10 µg/mL	3	3	3	3	3	3	3	3	3	YES
5 µg/mL	3	3	3	3	3	3	3	3	3	YES
Levamizole(LVZ)										
100 µg/ mL	3	3	3	3	3	3	3	3	3	YES
80 µg/mL	3	3	3	3	3	3	3	3	3	YES
40 µg/mL	3	3	3	3	3	3	3	3	3	YES
20 µg/mL	3	3	3	3	3	3	3	3	3	YES
10 µg/mL	3	3	3	3	3	3	3	3	3	YES
5 µg/mL	3	3	3	3	3	3	3	3	3	YES
Praziquantel (PZQ)										
3,0 µg/mL	–	–	–	–	–	–	–	–	–	NO

Score.

*0dead adults worms*

*1adults worms with marked reduction in motility and/or tegumental damage*

*2adults worms with reduced motility and/or tegumental damage*

*3adults worms without morphological changes (translucent) and with motility.*

*- evaluation suspended due to mortality in the previous timepoint*

Schistosomicidal activity of nitazoxanide (NTZ), praziquantel (PZQ), thiabendazole (TBZ), and levamisole (LVZ) against adult *Schistosoma mansoni in vitro*. NTZ exhibited pronounced, concentration- and time-dependent effects, inducing adult worm mortality at 100, 80, and 40 µg/mL from the first observation interval (24 h), while lower concentrations (20–5 µg/mL) impaired motility, inhibited oviposition, and induced tegumental alterations. PZQ showed comparable effects at the tested dose, whereas TBZ and LVZ did not produce significant motility reduction or morphological changes

### Nitazoxanide Treatment Causes Changes in the Ultrastructure of *Schistosoma mansoni*

**Fig. 2:**
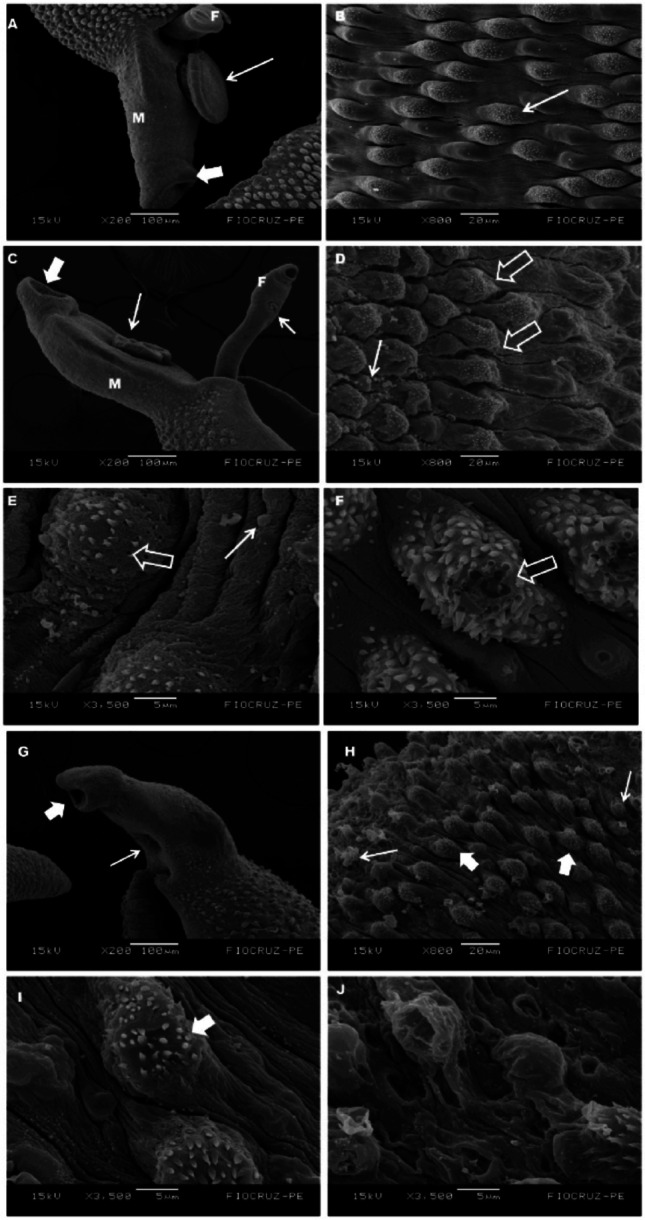
Ultrastructural evaluation by scanning electron microscopy of *Schistosoma mansoni* adult worms after treatment with nitazoxanide and praziquantel. *Note*: Scanning electron microscopy of *S. mansoni*. **A** Untreated male (M) and female (F) worms showing the pattern of ventral suckers (short arrow) and oral suckers (thin arrow). **B** Tegument of an untreated control male worm displaying well-arranged tubercles and spines (thin arrow). **C** Male (M) and female (F) worms 15 min after NTZ treatment exhibiting wrinkling of oral and ventral suckers. **A** Tegumental alterations in male worms after NTZ treatment, showing shortening of ridges near the tubercles (open arrow); some tubercles appearing smooth, and numerous small protrusions (thin arrow). **E** Male worm treated with NTZ presenting tegumental erosion and spine loss (open arrow), along with small protrusions (thin arrow). **F** Male worm treated with NTZ showing focal lesions on tubercles (open arrow). Scanning electron microscopy of male worms treated with PZQ. **G** Male worm treated with PZQ showing collapsed oral (short arrow) and ventral suckers (thin arrow). **H**- **I** Wrinkled tegumental ridges, tubercles with spine loss (short arrows), and vesicular protrusions (thin arrows) are observed. **J** Extensive and severe erosion and desquamation of the tegument in the male worm treated with PZQ

Based on the higher schistosomicidal effects of NTZ when compared to TBZ and LVZ, we performed an ultrastructural assessment of the parasite after NTZ treatment and compared the observed alterations with those induced by PZQ. Adults worms treated with NTZ (Fig. 2C-F) displayed significant morphological alterations, including wrinkling of oral and ventral suckers, tegumental changes in male worms, such as shortening of the tegumental ridges near tubercles, the appearance of smooth tubercles, and the formation of numerous small protrusions. In some cases, erosion and spine loss on the tegument were also observed, accompanied by focal lesions on tubercles. To directly compare drug effects on the parasite, an ultrastructural analysis was also performed for PZQ. This drug considered the gold standard for schistosomiasis treatment, induced parasite death, as illustrated in (Fig. 2G-J). PZQ treatment resulted in collapsed oral and ventral suckers, wrinkled tegumental ridges, spine loss, and the presence of vesicular protrusions. Furthermore, severe tegumental erosion and desquamation were observed in worms treated with PZQ.

### In vivo Treatment Efficacy and Parasitological Evaluation

Following the confirmed in vitro schistosomicidal effects of NTZ on both juvenile and adult worms, we proceeded with in vivo evaluation using an acute *S. mansoni* infection model. The therapeutic regimen assessed NTZ, individually and in combination with PZQ. After the euthanasia of the animals, treatment efficacy was estimated by the percentage of recovered worms, as summarized in Table 3. In groups treated with PZQ alone or in combination with NTZ, 100% efficacy was observed, as no worms were collected. In contrast, the efficacy of nitazoxanide alone was 57.14%.

**Table 3:** Evaluation of the efficacy of treatment with praziquantel (PZQ), nitazoxanide (NTZ) and the combination between them in animals infected with *Schistosoma mansoni* - acute phase

Drugs	Nº animals	Worms recovered		Percentage of treatment efficiency. reduction (%)=(C-T)/C x 100
		Nº total	Mean ± SD	
untreated	7	91	13.00 ± 6.56	-
PZQ 400 mg/kg	4	0	0.00 ± 0.00	100
NTZ 205 mg/kg	7	42	5.57 ± 4.96	57.14
Combination of PZQ 400 mg/kg + NTZ 205 mg/kg	5	0	0.00 ± 0.00	100

C= average number of worms per animal in the group of untreated animals

T= average number of worms per animal in the group of treated animals

**Fig. 3:**
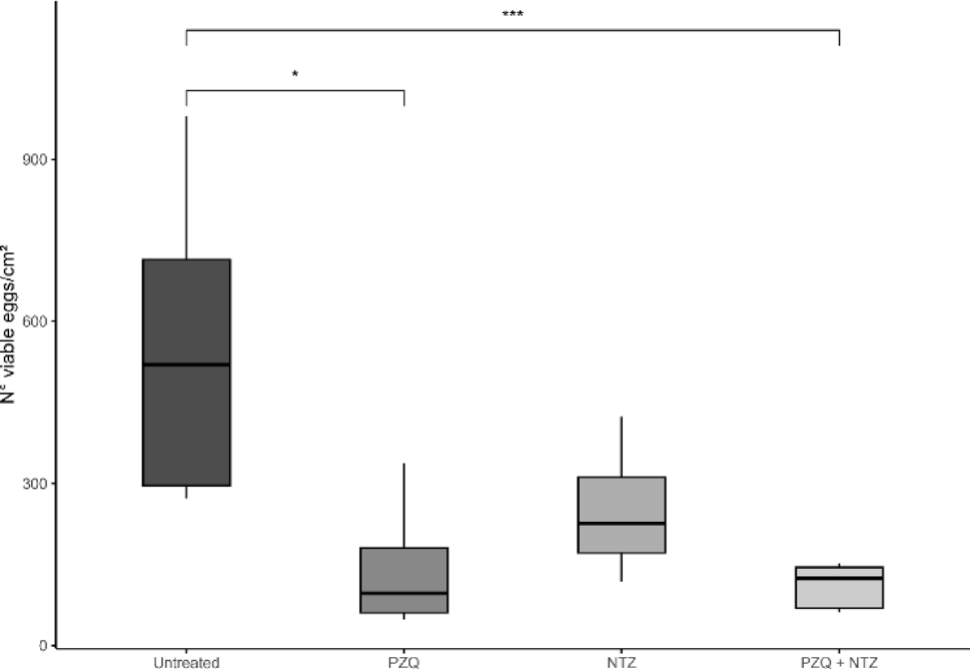
Oogram for verification of viable eggs of *Schistosoma mansoni* after treatment with PZQ, NTZ and the combination PZQ + NTZ *Note.* Boxplot showing the results of the oogram experiment. Data were analyzed using the Kruskal–Wallis test followed by Dunn’s post hoc test with Bonferroni correction. Significant differences were observed between the untreated group and the groups treated with PZQ, both alone and in combination between PZQ e NTZ. *p value* * 0.029; ***0.0025

To assess the ability of residual worms to continue releasing viable eggs post-treatment, the oogram technique was employed. The mean number of viable eggs obtained from infected/untreated animals was 541.86 ± 273.13 eggs/cm^2^. The groups treated with NTZ (205 mg/kg) or PZQ (400 mg/kg) exhibited mean values of 247,98 ± 109,68 and 144,92 ± 133,46 eggs/cm^2^, respectively. When NTZ and PZQ were administered in combination, the treatment resulted in a more pronounced reduction, yielding a mean of 96,75 ± 49,55 eggs/cm^2^ (Fig. 3).

These results confirms that PZQ is effective in reducing egg counts in infected animals and suggest that the combination of PZQ and NTZ may enhance therapeutic efficacy.

## Discussion

According to the results obtained here, the combination of NTZ and PZQ showed additional effects on egg viability in vivo, considering that NTZ administered alone did not produce effects superior to PZQ in the in vivo model. In contrast, TBZ and LVZ showed insufficient performance to induce parasite mortality in schistosomula or adult worms.

The findings regarding the lack of in vitro efficacy of levamisole and thiabendazole against *Schistosoma* did not support the progression to in vivo evaluations for these drugs. The absence of parasiticidal activity of levamisole observed here may be attributed to the requirement for higher drug concentrations or an extended assessment period. Similar results were reported by Campillo (2022) [63] in a study evaluating levamisole against another helminth, *Wuchereria bancrofti*, the etiological agent of filariasis, where only a transient reduction in parasite burden was observed. That study recommended further investigation of alternative dosing strategies, including higher drugs concentration or prolonged administration, to enhance an efficient therapeutic result.

Similarly to Karpstein (2025) [64], the 2025 study demonstrated that thiabendazole, tested at 50 µM, showed no in vitro schistosomicidal activity against schistosomula or adult *S. mansoni* worms, reinforcing the lack of therapeutic potential of this compound. EL Bialy et al. (2013) [65] assessed BTP-Iso, another benzimidazole derivative, and reported that at a dosage of 300 mg/kg, it exhibited schistosomicidal activity resulting in a significant reduction in parasite burden in vivo studies. Probst et al. (2021) [66] investigated novel benzimidazole derivatives, specifically pyrido[1,2-a] benzimidazole (PBI) and observed in vitro schistosomicidal activity against both NTS and adult worms. However, the therapeutic window was considered narrow, as in vivo results demonstrated only moderate worm reduction, ranging from 35.8% to 89.6% in high-parasite-load *S. mansoni* infections.

Biendl et al. (2022) [67] evaluated nitazoxanide in vitro, reporting schistosomicidal activity exceeding 66% after 72 h of drug exposure in both newly transformed schistosomula and adult *S. mansoni* worms (EC₅₀ = 0.47 ± 0.07 µM). In the present study, nitazoxanide demonstrated activity against both schistosomula and adult worms. At a concentration of 40 µg/mL (130 µM), NTZ induced 100% mortality of NTS, and a comparable effect was observed in adult worms, in which oviposition was completely halted across all tested concentrations. Moreover, when combined with PZQ, NTZ did not improve worm burden reduction compared to PZQ monotherapy but increased the proportion of non-viable eggs, underscoring a potential complementary effect on parasite reproductive output rather than superior overall efficacy.

Importantly, the concentrations required to achieve complete mortality of both schistosomula and adult worms in vitro (~ 130 µM) exceed the peak plasma concentrations (Cmax) of tizoxanide, the pharmacologically active metabolite of nitazoxanide, reported after standard oral dosing in humans. Nitazoxanide is rapidly hydrolyzed to tizoxanide, which represents the circulating active form, while the parent compound is not detectable in plasma [68]. Following a 500 mg oral dose, peak plasma concentrations of tizoxanide are typically in the low micromolar range (approximately 1.9–2.0 µg/mL ~ 6–7 µM) [69]. In addition, tizoxanide exhibits high plasma protein binding (> 99%) [70], which may further limit free systemic exposure. Although systemic plasma concentrations may not directly reflect drug levels at sites relevant to parasite biology such as the intestinal lumen and portal-hepatic compartments, these pharmacokinetic characteristics should be considered when interpreting the in vitro concentrations required for schistosomicidal activity.

In the present murine model, administration of 205 mg/kg, corresponding to the human equivalent dose adjusted by body surface area scaling [60], resulted in a 57% reduction in parasite burden. It should be noted that the relatively small sample in the in vivo groups may limit statistical power. animal numbers were defined according to ethical principles, the 3R’s guidelines, with the use of inbred mice, however, some animals were lost during the course of the experiment, resulting in a reduced final sample size. Additional studies involving larger cohorts will be valuable for expanding upon and further characterizing these findings.

The ultrastructural alterations observed after treatment with nitazoxanide indicate that this drug progressively compromises the tegumental integrity of *S. mansoni*, suggesting a distinct and less aggressive mechanism of action when compared to praziquantel. Wrinkling of the suckers, shortening of tegumental ridges, and the presence of protrusions and focal lesions suggest structural dysfunction and a potential impairment of parasite adhesion, nutrition, and host–parasite interactions, which may contribute to reduced parasite viability. In contrast, the more extensive damage induced by praziquantel, including sucker collapse, severe erosion, and tegumental sloughing, reflects its well-established rapid and intense effect on the tegument, as described by Filho et al. (2020) [71]. Thus, although nitazoxanide exhibits lower schistosomicidal activity than praziquantel, the induced morphological alterations reinforce its potential as a complementary or repositioned agent, particularly considering its impact on tegumental integrity and the possibility of use in adjunctive therapeutic strategies.

El-Taweel et al. (2016) [34] compared the ultrastructural effects of nitazoxanide and praziquantel on *Schistosoma mansoni* recovered from animals two weeks after treatment with NTZ (100 mg/kg) and reported tegumental damage in both male and female worms, although concluding that NTZ exhibited lower efficacy than PZQ. In addition to corroborating these findings in adult worms, our results expand this evidence by demonstrating that NTZ also affects juvenile stages and significantly impairs oviposition, indicating a broader impact on the parasite life cycle. While El-Taweel et al. 2016 [34] performed scanning electron microscopy on worms recovered from animals after treatment, our study employed direct in vitro exposure at a sublethal concentration (10 µg/mL) for a short period (15 min), allowing the assessment of early drug-induced damage. This experimental approach suggests that NTZ can rapidly compromise tegumental integrity, reduce reproductive output, and interfere with parasite development, reinforcing its potential role as a complementary repositioned agent, particularly in strategies targeting both immature forms and egg production.

Our findings suggest that the combination of PZQ and NTZ does not increase parasite burden reduction beyond that achieved by PZQ alone, but significantly reduces viable egg counts, potentially due to an additive interaction between the two compounds. NTZ may augment the host immune response, while PZQ directly exerts schistosomicidal activity against the parasites. Although praziquantel has been extensively demonstrated to be effective against *S. mansoni*, our findings suggest that its combination with nitazoxanide may provide complementary benefits related to egg viability and transmission dynamics, particularly in the context of reinfections. Further studies are required to evaluate the long-term effects and optimize combination therapy in endemic regions.

Praziquantel remains the cornerstone of schistosomiasis treatment due to its well-established efficacy, favorable safety profile, and low cost, which has enabled its widespread use in large-scale control programs in endemic regions [72]. Although praziquantel is generally well tolerated, mild and transient adverse effects may occur, most commonly including abdominal discomfort, dizziness, headache, nausea, and fatigue, which are often associated with the host immune response to dying parasites rather than direct drug toxicity [73]. Nitazoxanide, in contrast, is a broad-spectrum antiparasitic drug widely used for the treatment of several protozoal infections and is also considered safe and well tolerated in humans [74]. The most commonly reported adverse effects associated with nitazoxanide are mild and transient and mainly include gastrointestinal symptoms such as nausea, abdominal discomfort, and diarrhea, as well as occasional headache [75]. Although nitazoxanide is commercially available in many countries, its cost and accessibility may vary depending on healthcare systems and drug formulations [26, 74].

Hu et al. (2013) [76] concluded that the efficacy of nitazoxanide or any other pharmacological agent against a pathogen depends on achie6ving and maintaining therapeutic concentrations for a sufficient duration to induce physiological alterations necessary for treatment success. Despite the promising findings of this study, certain limitations should be acknowledged. The experimental model was limited to murine hosts, which may limit the direct translatability of the results to humans. Furthermore, the post-treatment follow-up period was relatively short, precluding the evaluation of the long-term effects of the combination therapy. Nevertheless, our findings provide novel and relevant insights into potential therapeutic alternatives for the treatment of schistosomiasis in endemic regions. The combination of nitazoxanide and praziquantel may represent a complementary strategy aimed at reducing egg viability, rather than improving adult worm elimination.

## Conclusion

Based on the results of this study, the combination of nitazoxanide (NTZ) and praziquantel (PZQ) may represent a complementary approach for improving egg-related outcomes in *Schistosoma mansoni* infection, whereas thiabendazole (TBZ) and levamisole (LVZ) did not exhibit sufficient efficacy to induce parasite mortality in schistosomula or adult worms. Notably, this study demonstrates, for the first time, the schistosomicidal activity of NTZ against juvenile stages of *S. mansoni*, while also providing evidence of marked structural alterations in adult worms. Although these findings do not support NTZ as a standalone therapeutic option, its activity against early parasite stages and its impact on adult worm integrity emphasize its potential relevance as an adjunctive agent, particularly in endemic settings where continuous exposure favors reinfection. In this context, drugs capable of targeting immature stages may provide important complementary benefits. Therefore, further studies are necessary to investigate the effects of NTZ and PZQ co-administration across different stages of *S. mansoni* infection and to elucidate its impact on egg viability, transmission dynamics, and long-term disease progression.

## Data Availability

The data supporting the findings of this study are available within the article.
